# Commonization of HIV/AIDS services in Nigeria: the need, the processes and the prospects

**DOI:** 10.11604/pamj.2014.19.329.5138

**Published:** 2014-11-27

**Authors:** Obinna Ositadimma Oleribe, Olabisi Oladipo, Princess Osita-Oleribe, Chukwuemeka Nwachukwu, Frank Nkwopara, Ekei Ekom, Solomon Nwabuzor, Grace Iyalla, Kenneth Onyewuchi, Ayodotun Olutola, Okey Nwanyanwu, Peter Nsubuga

**Affiliations:** 1 Excellence and Friends Management Care Centre (EFMC), Gwarimpa, Abuja, Nigeria; 2 Brandeis University, MA, Waltham, Massachusetts, USA; 3 Center for Clinical Care Research, Nigeria (CCCRN); 4 Global Health Services Network LLC, Michigan, USA; 5 Global Public Health Solutions Atlanta GA, USA

**Keywords:** Human immunodeficiency virus, acquired immune deficiency syndrome, integration, sustainable, commonization, decentralization

## Abstract

**Introduction:**

With the first case of Human Immunodeficiency Virus infection/Acquired Immunodeficiency Syndrome (HIV/AIDS) identified in 1986, the management of HIV/AIDS in Nigeria has evolved through the years. The emergency phase of the HIV/AIDS program, aimed at containing the HIV/AIDS epidemic within a short time frame, was carried out by international agencies that built structures separate from hospitals’ programs. It is imperative that Nigeria shifts from the previous paradigm to the concept of *Commonization* of HIV to achieve sustainability. *Commonization* ensures that HIV/AIDS is seen as a health condition like others. It involves making HIV services available at all levels of healthcare.

**Methods:**

Excellence & Friends Management Consult (EFMC) undertook this process by conducting HIV tests in people's homes and work places, referring infected persons for treatment and follow up, establishing multiple HIV testing points and HIV services in private and public primary healthcare facilities. EFMC integrated HIV services within existing hospital care structures and trained all healthcare workers at all supported sites on HIV/AIDS prevention, care and treatment modalities.

**Results:**

Commonization has improved the uptake of HIV testing and counseling and enrolment into HIV care as more people are aware that HIV services are available. It has integrated HIV services into general hospital services and minimized the cost of HIV programming as the existing structures and personnel in healthcare facilities are utilized for HIV services.

**Conclusion:**

Commonization of HIV services i.e. integrating HIV care into the existing fabric of the healthcare system, is highly recommended for a sustainable and efficient healthcare system as it makes HIV services acceptable by all.

## Introduction

With a national HIV prevalence of 3.4% as at 2012, HIV/AIDS is one of commonest causes of death in Nigeria [1]. It is estimated that 3,400,000 adults and children in 36 states and the Federal Capital Territory (FCT) of the Federation are living with HIV with about 260,000 new infections occurring annually [2]. Although the epidemic is now described as stable as a result of its prevalence hovering around 4% in the last five years [3], Nigeria has lost hundreds of thousands of bread winners to this epidemic. Over the past decade, there has been an unprecedented global response to the AIDS epidemic leading to a rapid scale-up of HIV treatment and prevention which has consequently saved millions of lives and transformed communities around the world. Since 1999, the year in which the epidemic is thought to have peaked globally, the number of new infections has decreased by 19% [4]. The lowest levels of new HIV infections have been reported this century at 2.1 million. In the last three years alone, new HIV infections have fallen by 13% [5]. More than 2.5 million deaths have been averted through treatment since 1995, and currently 6.6 million people are receiving treatment in low-income and middle-income countries [4]. Since 2004, when treatment of HIV/AIDS started effectively in Nigeria, it has been largely donor dependent. In 2007, 85.4% of all HIV expenditure was derived from external sources and this increased to 92.35% in 2008 [6]. Through these programs, 538,000HIV-infected people had received Anti-retroviral therapy (ART) as at 2013 [7]. However, the goal of universal access is limited by number and location of service delivery points. As at 2013, only 6,675,000 people were tested for HIV [7] and this represents about 4% of the total population of Nigeria while, only 17% of HIV positive women received Anti-retroviral (ARV) drugs for Prevention of Mother-to-Child Transmission (PMTCT) [8]. In existing treatment sites, HIV services are provided through vertical programs with specialized personnel, separate laboratories and specific clinic days. This programming model is expensive, unsustainable and fuels stigma and discrimination among HIV-infected and affected persons.

As demand for HIV treatment and care services increases, the health systems as currently supported will be increasingly stretched while resources available through donor agencies remain constant or decline. Therefore, there is the need to expand the capacity of health systems and personnel in a sustainable manner, compatible with existing mechanisms for managing chronic illnesses. Subject matter experts opine that key strategies that should be employed to sustain HIV treatment and care programs in high HIV-prevalence low and middle-income countries (like Nigeria) over the coming decade include further decentralization, task shifting, and integration of HIV services with other chronic disease treatment services [9]. In the long-term, the increased demand for HIV-care services can only be satisfied through increased decentralization to peripheral health units, with the role of each type of unit being appropriate to the human and material resources available to it [10]. Integration of HIV services is defined as co-location and sharing of services and resources for HIV care and primary care, such as clinic space, clinicians, health education, pharmacy, laboratory services, and training [11].

As a result of non-integration of HIV services into the fabric of the healthcare systems at all healthcare facilities, once there is cessation of funding by donor agencies, a collapse of the HIV program will result. However, this highlights the need for realistic budgeting by national governments [9]. While these strategies address the supply end of the HIV service chain, the demand can only be addressed by increasing client confidence through improved understanding of HIV and concerted efforts in eliminating stigma and discrimination. Despite the current decentralization, there is little evidence for improving access and adherence among vulnerable groups such as women, children and adolescents, and other high-risk populations and for addressing major barriers [12]. To address this, reduce cost of management and ensure sustainability of care – even post-funding era, Excellence and Friends Management Consult (EFMC) conceptualized *commonization* as a key necessity to a world free of HIV and AIDS [13]. The *commonization* of HIV services comprises, but is not limited to, decentralization and availability of services where people live and work, training of all healthcare workers to conduct daily clinics for HIV-infected patients as well as patients with other morbidities, thus, fully integrating HIV services into the fabrics of the hospital system. It also emphasizes that HIV should not be perceived as a special disease but like any other chronic ailment.

## Methods

With funding from US Government, the need to reach more in a unique project Christened “Reaching All with Care and Support Services in HIV/AIDS (REACH), understanding of a future changing funding climate and desire to do more with less, Excellence & Friends Management Consult (EFMC) developed an innovative HIV programming matrix which fully integrates HIV services into the core health services of supported facilities. This new model dismantled parallel HIV programs existing in sites experienced in HIV service provision and refused to fund such structures in newly activated sites. This approach also required no special task force on HIV within the supported facility, no special HIV clinic/specific HIV clinic days, no special HIV laboratory, no special HIV pharmacy and no special HIV personnel.

In all EFMC-supported sites, prior to startup of HIV services, EFMC carried out a detailed assessment of the healthcare facilities using standardized assessment tools. This assessment covered all areas of health care service provisions including available manpower, infrastructure, and linkages to other medical services, among others. Following the assessment, EFMC's National Trainers, with support from relevant State agencies, conducted four to five-day on-site orientation for all relevant staff (doctors, nurses, pharmacists, laboratory staff, medical records staff) on all aspects of HIV care and treatment using standardized National Guidelines and curriculum required for effective service delivery. Post-training, EFMC provided on-the-job mentoring to all staff of the facility, job aids for every participating unit, data collection and reporting tools, as well as drugs, consumables, computers and laboratory equipment. All equipment were installed in the general hospital laboratory and used for all hospital clients. Drugs were stored in the central pharmacy and records were kept in the hospital records section. Also, patients were seen every day of the week in all out-patient clinics, HIV testing was done at the laboratory, consulting rooms, counseling sections, ante-natal clinics and even in stand-alone sites. EFMC facility facilitators supported the facility adoption of the process, advocated for Provider-Initiated HIV testing and counseling (PITC), ensured quality of services and provided skilled support in areas of human resources for health challenges. To ensure facility ownership of the entire project, a project management team headed by a senior clinician was inaugurated in all facilities and supported to meet monthly to review successes recorded as well as manage challenges encountered. To enhance the reach of the project, community demand creations were initiated and sponsored and all reactive clients referred to the comprehensive ART sites.

To achieve sustainability and ownership of the HIV program by healthcare workers, all supported sites were encouraged to own their data, use their data to develop manuscripts as well as inform program review, strategy and improvement activities. Weekly reports of activities were required from all supported sites. Monthly meetings of PLHIV Support groups were encouraged and established. During these meetings, both clients and facilities’ staff interact to discuss issues relating to their wellbeing and sustainable ways of managing HIV/AIDS and its effect.

## Results

**Figure 1:**
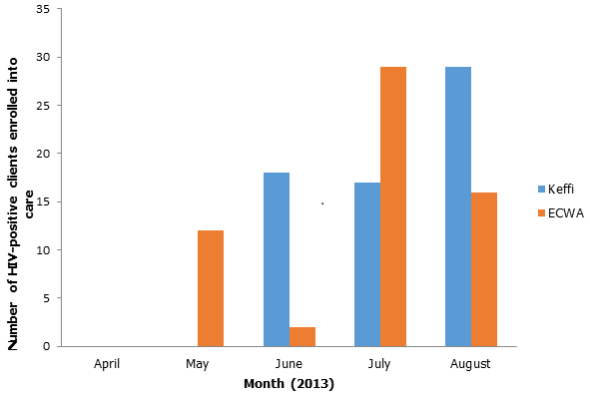
Enrolment per month at General Hospital, Keffi and ECWA CHC, Karu following training of all healthcare workers at the hospitals in May 2013

One hundred and twenty-one public and private healthcare facilities were engaged during this process over 18 months (from October 2011 to March 2013) in Federal Capital Territory (FCT), Nasarawa and Imo States of Nigeria. HIV services were fully integrated into the routine services at the facilities with the same personnel and infrastructures used for HIV positive and non-positive clients. Facilities which were naïve to HIV care and treatment adapted to the new programming much faster than sites experienced in HIV programming as it was more challenging to get the staff of the experienced facilities to buy in to the concept of *commonization* because they were used to the previous programming paradigm. As HIV care and services were demystified and relevant knowledge shared freely among health workers, health systems improved as previously disenfranchised health workers had good access to HIV knowledge. This resulted in provision of better quality of care to HIV-infected persons as the hospital staff were being carried along in the trainings on patient management. In addition, weekly enrolment of HIV-infected patients into care at the facilities improved by more than 200% (Figure 1) and reports were received on time. Overall, the cost of programming reduced significantly by an average of 45% as the same personnel and infrastructure were used for HIV-positive and general hospital patients. The HIV-infected clients expressed delight that the stigma and discrimination previously experienced by them as a result of having special clinic days and separate rooms for consultation had been eliminated. They were free to walk into any part of the facilities without being tagged [13].

## Discussion

Knowing the HIV status of every Nigerian is the first step towards controlling HIV in the country and HIV testing and counseling is a gateway for access to comprehensive HIV services including Anti-retroviral and Prevention of Mother-to-Child Transmission (PMTCT) services. There is increasing recognition that the essential ′package′ of HIV care must include early identification of HIV-positive people in need of care through testing, appropriate initial and continued counseling, assessment of HIV disease stage, treatment with Anti-retroviral Therapy for those who need it, monitoring while on treatment for efficacy, adherence and side-effects, detection and management of other complications of HIV infection, provision of sexual and reproductive health services as well as careful record-keeping. The impressive scale-up of HIV treatment and care services has required decentralization of service provision linked to task-shifting. But the future holds even greater challenges, as the number of people in need of HIV care continues to rise at a time when many traditional donors and governments in the most-affected regions have reduced budgets [14]. The study carried out by Tran and Nguyen in 2012 highlights the importance of improving the quality of HIV/AIDS services at the provincial and district clinics. Potential strategies recommended include capacity building for health workers, integrative service delivery, engagements of family members in treatment supports, and additional attention and comprehensive care for drug users with HIV/AIDS [15].

Over the past nine years of HIV/AIDS programming, most implementing partners have focused on providing services through standard health facilities. However, this has reduced access, minimized reach and kept millions of Nigerians ignorant of their HIV status thereby preventing their accessing care and support services. As HIV programming moves from the emergency mode to sustainability mode, full integration through effective *commonization* of services is critical. New programming strategies developed and implemented by EFMC to reach the large number of previously unreached people include provider-initiated testing and counseling, multi-point facility based testing, mobile testing, community level testing, house-to-house and shop-to-shop testing.

**Figure 2:**
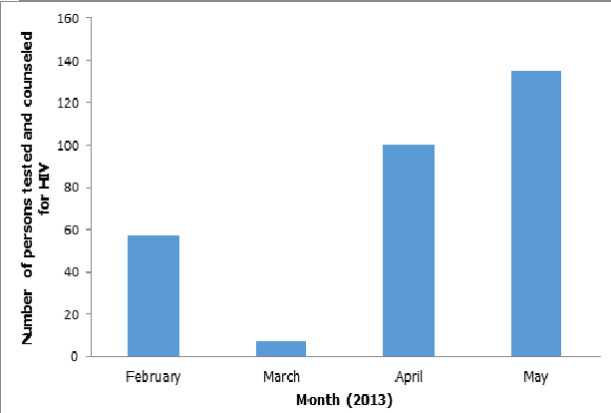
Increase in HTC during Immunization clinics after integration of HIV services at PHC Lugbe (February to May 2013)

In 2007, the World Health Organization (WHO) recommended provider-initiated HIV testing in health care facilities as a standard part of medical care in generalized HIV epidemics, intending to expand current practices of client-initiated voluntary counseling and testing (VCT). Provider-initiated testing capitalizes on all patient contacts with the medical system, using each as a potential opportunity for HIV testing, diagnosis, and linkage to care [16]. EFMC has employed this strategy whereby every healthcare worker who comes in contact with a patient for whatever reason offers the patient HIV testing and counseling with room to opt-out, if need be. Studies in various African countries have shown that provider-initiated testing integrated in routine health care is acceptable to patients, increases testing participation, and may identify HIV-infected individuals at an earlier disease stage [16]. With the institution of HIV screening in hospitals and emergency departments, the percentage of patients who tested positive in that study ranged from 2 to 7% and this exceeded that observed nationally at publicly funded HIV counseling and testing sites (1.5%) [17]. This strategy has been expanded by EFMC to make available multiple testing points at healthcare facilities (such as in the emergency units, wards, OPD clinics, immunization clinics, STI clinics, etc). This has made access to testing and counseling services much better as people referred for HIV tests can have them done on the spot, without having to move long distances to central laboratories, giving room to high chances of default. Figure 2 shows the increase in HTC after integration of HIV services into the Immunization clinic at PHC Lugbe, one of EFMC's supported sites.

In a study carried out among TB cases in West Bengal, India, it was concluded that comprehensive strategies to change providers′ beliefs and practices, decentralization of HIV testing to all TB care centers, and improved HIV test kit supply chain management may increase the proportion of patients with TB who are tested for HIV [18].

HIV-care services can also naturally integrate with the care of chronic non-communicable diseases and with closely related services like mother and child health, and thus should promote a shift from vertical to integrated programming. Staff training and support around a set of evidence-based policies and guidelines and a reliable supply of essential medicines and supplies are further essential components for a successful program [10].

Full integration of HIV services into facilities’ health services is critical for the realization of an AIDS-free generation. This can be achieved by training all healthcare workers on HIV management, making HIV services common at all levels so as to enhance effectiveness and efficacy. HIV infection should not be perceived as a “special” disease, requiring special personnel, infrastructure, resources, clinic days, etc. Rather, integration of HIV services into the fabric of the general healthcare system should be implemented to improve resource utilization, improve quality of services and eradicate stigma and discrimination. In a study carried out in Kenya by Odeny et al., it was observed that patient education sessions on HIV had a greater impact and were more beneficial because they were done in an integrated setting where there was a mixed population of HIV-positive patients and general patients. There was also increased satisfaction with patient reception by hospital staff, reduced waiting times and improved patient-provider interactions as a result of training of all healthcare workers on HIV services [19].

Mobile testing, community level, house-to-house and shop-to-shop testing are also vital in *commonizing* HIV in that there is increased awareness, and thus, demystification of the infection. These modalities ensured that persons who conventionally would not visit health care centers were reached and tested for HIV. In order to empower Nigerians with knowledge of their HIV status, testing people where they live and work is an effective mechanism. People will access services if brought closer to them at free or cheaper rate. A study in Malawi showed that household members in the lowest-income quartile were significantly less likely to have ever used facility-based HIV testing than the rest of the population but were 70% more likely to use the home-based rapid HIV testing program [20]. Most Nigerians, whether in urban or rural settings, are aware of the importance of immunization and this is as a result of the massive campaigns and outreaches carried out in communities. This process has been employed in National Immunization days and can also be replicated to HTC, as a national strategy.

In addition to the above strategies to de-centralize HIV services and provide services where people live and work, engagement of Primary Healthcare facilities and Private Medical Vendors is a viable tool to achieve this. More than 48% of Nigerians seek health care from Private Medical Vendors (PMVs) while about 32% of the population goes to Primary Healthcare Centers (PHCs). Thus, only about 20% of Nigerians access care at public secondary and tertiary centers [21]. The PMVs and PHCs are healthcare facilities which are, most times, located short distances to where people live and work. It is, therefore, logical to make HIV services available at such facilities that are within reach of members of communities for easier access. This will also assist in demystifying the concept of HIV and HIV care as far-reaching, abstract and inaccessible to the common man. Having HIV care services available at these facilities in addition to the public secondary and tertiary healthcare facilities, significantly improves uptake, ensures prompt initiation of ART among patients, reduces loss to follow up (especially among pregnant women) and achieves greater geographic HIV care coverage [22].

Certain challenges were encountered while trying to implement this concept of *commonization* at some of the facilities EFMC worked with. There was non-acceptance of the *commonization* concept by some healthcare workers as they having been used to receiving special stipends while working in the vertical HIV programs set up by donor agencies. Introducing the *commonization* concept to them, while other facilities still run standalone HIV programs and receive special stipends gives room for low interest in the concept. In order to achieve sustainability, there should be government backing to train all healthcare workers, not a few, on HIV/AIDS management thus, emphasizing that the disease is like any other so such funds have to be stopped across board.

The mindset whereby healthcare workers perceive the management of HIV/AIDS as extra burden to their normal duties is yet another challenge. But with wide acceptance of the *commonization* concept, this mindset will change, especially if national Governments are fully involved and emphasize that HIV/AIDS management is an integral part of normal hospital duties.

More so, the number of tools designed to monitor the HIV/AIDS program, which is seen by some healthcare workers as too many, compared with what is obtainable with the management of other disease conditions is also a challenge to integrating HIV/AIDS program into the general healthcare system. Also, religious beliefs have made people perceive HIV/AIDS as a spiritual issue that needs to be handled spiritually thereby negating the concept of *“commonization”*.

## Conclusion

HIV/AIDS is a chronic infection that has hitherto been regarded as a special disease resulting from the approach international donor agencies (which have funded the HIV/AIDS program up till this time) have used to manage it. In the emergency phase of the HIV/AIDS epidemic, there was need to set up structures, employ skilled professionals specifically for its management and these were separate from general hospital services. This was done in order to achieve rapid results. However, this approach has fuelled stigma and discrimination of HIV-infected clients, disenfranchisement of health workers not involved in the HIV program, limited persons in the general population from seeking to know their HIV status due to inadequate knowledge and fear of discrimination, among others. The approach of *commonizing* HIV/AIDS and making it no different from any other infection or health condition seen in the healthcare facilities through conduct of HIV tests at all levels of hospitals, multiple points within hospitals, dismantling of HIV-specific structures and special HIV clinic days is very important as this will help to improve uptake of HIV/AIDS, reduce discrimination of clients and empower all healthcare workers in the management of HIV/AIDS. *Commonizing* HIV/AIDS is critical to achieving an AIDS-free generation.

## References

[1] Federal Ministry of Health (Nigeria) (2013). National HIV&AIDS and Reproductive Health Survey, 2012.

[2] UNAIDS Report on the Global AIDS Epidemic-2013. http://www.unaids.org/en/dataanalysis/knowyourepidemic.

[3] The President's Comprehensive Response Plan (PCRP) http://www.zero-hiv.org/wp-content/uploads/2013/07/PCRP-Summary.pdf.

[4] Kanki P, Kakkattil P, Simao M (2012). Scaling up HIV treatment and prevention through national responses and innovative leadership. J Acquir Immune DeficSyndr.

[5] UNAIDS The Gap Report-2014. http://www.unaids.org/en/resources/presscentre/pressreleaseandstatementarchive/2014/july/20140716prgapreport/.

[6] UNAIDS (2011). Country Case Study: Nigeria. http://www.unaids.org/en/media/unaids/contentassets/documents/pcb/2011/20110614_CRP2_Nigeria%20PCB%20case%20study.pdf.

[7] PEPFAR Report Tenth Annual Report to Congress on PEPFAR:Direct FY2013 Testing and Counseling Results. http://www.pepfar.gov/press/222834.htm.

[8] World Health Organization (2013). (WHO) Report. Global Update on HIV Treatment 2013: Results, Impact and Opportunities.

[9] Ross DA, South A, Weller I, Hakim J (2012). HIV treatment and care systems: the way forward. AIDS.

[10] Munderi P, Grosskurth H, Droti B, Ross DA (2012). What are the essential components of HIV treatment and care services in low and middle-income countries: an overview by settings and levels of the health system?. AIDS.

[11] Tran BX, Nguyen NP (2012). Patient satisfaction with HIV/AIDS care and treatment in the decentralization of services delivery in Vietnam. PLoS One.

[12] Scanlon ML, Vreeman RC (2013). Current strategies for improving access and adherence to antiretroviral therapies in resource-limited settings. HIV AIDS (Auckl).

[13] Oleribe OE, Nwachukwu CE, Akande SF (2013). An AIDS-free world through the full decentralization of HIV services: a proof-of-concept study. The Lancet.

[14] Munderi P, Grosskurth H, Droti B, Ross DA (2012). What are the essential components of HIV treatment and care services in low and middle-income countries: an overview by settings and levels of the health system?. AIDS.

[15] Tran BX, Nguyen NP (2012). Patient satisfaction with HIV/AIDS care and treatment in the decentralization of services delivery in Vietnam. PLoS One.

[16] Basset IV, Walensky RP (2010). Integrating HIV screening into routine healthcare in resource-limited settings. Clin Infect Dis.

[17] CDC (2006). Revised Recommendations for HIV testing of Adults, Adolescents and Pregnant Women in Healthcare settings, 2006 MMWR.

[18] Bishnu B, Bhaduri S, Kumar AM (2013). What are the reasons for poor uptake of HIV testing among patients with TB in an Eastern India District?. PLoS One.

[19] Odeny TA, Penne J, Lewis-Kulzer J (2013). Integration of HIV Care and Primary Health Care Services: Effect on patient satisfaction and stigma in rural Kenya. AIDS Res Treat.

[20] Hellenringer S, Kohler HP, Frimpong JA, Mkandawire J (2009). Increasing uptake of HIV testing and counseling among the poorest in sub-Saharan countries through home based service provision. J Acquir Immune Defic Syndr.

[21] Oleribe OE, Akande F, Osita-Oleribe P (2013). Expanding PMTCT services to PHCs and Private Medical Vendors.

[22] Pfeiffer J, Montoya P, Baptista AJ (2010). Integration of HIV/AIDS services into African primary healthcare: lessons learned for health system strengthening in Mozambique-a case study. J Int AIDS Soc.

